# Implementation of an unconscious bias course for the National Research Mentoring Network

**DOI:** 10.1186/s12909-022-03466-9

**Published:** 2022-05-21

**Authors:** Damaris Javier, Linda Grace Solis, Mirabelle Fernandes Paul, Erika L. Thompson, Grace Maynard, Zainab Latif, Katie Stinson, Toufeeq Ahmed, Jamboor K. Vishwanatha

**Affiliations:** 1 Institute for Health Disparities, University of North Texas Health Science Center, 3500 Camp Bowie Blvd, Fort Worth, TX 76107 USA; 2 University of the Incarnate Word School of Osteopathic Medicine, San Antonio, USA; 3 Western University of Health Sciences, United States and College of Osteopathic Medicine of the Pacific-Northwest, Lebanon, Oregon USA; 4 Department of Biostatistics and Epidemiology, University of North Texas Health Science Center, Fort Worth, USA; 5 Vanderbilt University Medical Center, Nashville, USA

## Abstract

**Purpose:**

Increased awareness and mitigation of one’s unconscious bias is a critical strategy in diversifying the Science, Technology, Engineering, Mathematics, and Medicine (STEMM) disciplines and workforce. Greater management of unconscious bias can enhance diverse recruitment, persistence, retention, and engagement of trainees. The purpose of this study was to describe the implementation of an asynchronous course on unconscious bias for people in STEMM. Specifically, we explored who engaged with the course and reflections from participation.

**Method:**

A five-part, asynchronous Unconscious Bias Course was developed and was hosted on a national mentoring platform starting in July 2020. To examine course engagement, we assessed the demographics of course participants and completion. Participant responses to reflection questions after each module were also synthesized using qualitative methods.

**Results:**

Overall, 977 people registered for the course and 42% completed all modules. In the reflection responses, participants reflected on their unconscious biases in their lived experiences and how it relates to actions, judgements, external factors, stereotypes, and un-intentionality. Participants also reflected on microaggressions, their impact on the recipients and others, and the relationship between microaggressions and unconscious bias. Participants reported four key strategies used by allies against unconscious bias: immediately acting (83%), reflection (46%), improving the organizational culture (30%), and individual-level ally-ship (44%). Strategies for self-awareness included: reflection, pausing/breathing, and self-observation.

**Conclusion:**

The assessment of the Unconscious Bias Course implementation revealed the course reached a wide cross-section of people in STEMM and demonstrated that participants were able to reflect on the underpinnings of the course. This course, and its suite of offerings, support a nationwide effort to mitigate bias and prepare individuals to be culturally competent in a diverse society in order to foster a STEMM environment that caters to individuals’ success and diversification of these fields.

## Introduction

Diversifying the Science, Technology, Engineering, Mathematics, and Medicine (STEMM) workforce is of utmost importance to produce scientific and technological innovation and reap the benefits of heterogeneous diverse groups [1–3]. Furthermore, as the U.S. becomes more diverse, it is necessary to prepare individuals to become productive democratic citizens in an increasingly pluralistic nation and world. Unfortunately, unconscious bias is a cause of discrimination and structural racism, and an impediment to individuals’ access, wellness, persistence, and success in the STEMM fields [4, 5]. Research has highlighted that many of our biases are unconscious rather than conscious [6–9]. Unconscious bias is a prejudice we have or an assumption we make about another individual based on common cultural stereotypes, rather than on thoughtful judgment [10, 11]. Unconscious bias affects those in STEMM fields in many ways. Unconscious bias may hinder an individual’s ability to enter or pursue the field [5]. The extent of unconscious bias within admissions committees has been shown to impede an individual’s entrance to medical school [12–15]. For instance, in a study examining bias within admissions committees, the authors found that after taking the Implicit Association Test, all members of the committee comprising women, men, faculty, and students displayed significant white preference, with faculty having the largest bias [12].

Biases can affect hiring practices and the learning environment. Word of mouth is considered one of the most used tactics in recruiting and hiring employees, and favors job seekers who identify as white [9]. Furthermore, search committees, hiring staff and faculty, can also be influenced by unconscious bias held by members impeding the diversification of faculty and staff [14, 16, 17]. Unconscious bias can also affect students’ learning environment in several negative ways. Students of color are especially impacted by stereotyping from professors and administrators as well as students’ self-perceptions [11]. Unconscious bias also impacts students’ true integration and engagement within learning environments, which results in a lower sense of belonging at an institution [18]. Moreover, implicit bias can impact learner assessment due to subjectivity in grading and instructor bias [19, 20].

Strategies have been identified to overcome systemic barriers to the inclusion of underrepresented groups in science, one of which is anti-bias training [21]. Despite these recommendations for addressing systemic barriers, there is current debate about the efficacy of diversity and bias training as an intervention to change behavior [22–24]. In 2020, the National Research Mentoring Network (NRMN) built the NRMN Unconscious Bias Course, freely available to members in STEMM. The NRMN, funded by the National Institutes of Health, is a national program that provides individuals across all career stages in the biomedical, behavioral, clinical, and social sciences with evidence-based culturally responsive mentoring, networking, and professional development [25–27]. The NRMN program, currently with over 20,000 participants, reaches mentors and mentees across the U.S. and provides an ideal platform for engaging with, and providing resources to, a large group of mentees and mentors who are are participating in the mission to diversify the STEMM research workforce. The purpose of this study was to describe the implementation of an asynchronous course on unconscious bias for people in STEMM. Specifically, we explored who engaged with the course and reflections from participation.

## Method

The NRMN team developed and implemented an asynchronous online course focused on unconscious bias and strategies to overcome unconscious bias. The development team included experts in bias (Dr. Mirabelle Fernades Paul, Dr. Linda Grace Solis); instructional designers (University of North Texas Health Science Center’s Division of Academic Innovation) provided expertise in developing online course modules for NRMN’s virtual community, MyNRMN [25].

The Unconscious Bias Training content was developed by Dr. Fernandes Paul and Dr. Solis who have delivered diversity training sessions at their respective institutions. The purpose of the course was to describe implicit bias and the relationship between individual bias and existing disparities in healthcare. The content was informed by the perspective of bias by Howard J. Ross, including the concepts of awareness of unconscious bias, what happens after awareness, workplace culture, and personal and organizational ability to change [28]. The target audience for the course included individuals across the healthcare continuum (e.g., medical students through attending physicians) and students/researchers in the basic sciences.

The NRMN Unconscious Bias Course was finalized in July 2020 with five modules: Unconscious Bias, Microaggressions, Solutions Toolkit, Self-awareness, and Bias and Disparities in Healthcare. Table 1 lists the module topics and objectives. The NRMN Unconscious Bias Course may be completed all at once, or each module may be taken separately. A certificate is awarded upon completion of all modules. Each module takes approximately 20 minutes to complete. The NRMN Unconscious Bias Course has been leveraged in two main ways: (1) individuals self-selecting to take the course for their own development in regard to unconscious bias, (2) our partners, which include organizations, institutions/colleges, and professional/scientific societies, assign the course to their community of students, staff, and faculty. Within the MyNRMN platform, our partners can utilize the My Cohort feature, allowing them to create a course cohort for their members and to track metrics. The NRMN Unconscious Bias Course was released on July 17, 2020, and as of October 18, 2021, 1501 individuals enrolled in the course.

**Table 1:** Module topics and objectives for unconscious bias course

Module	Overview	Module Learning Objective
Module 1: Unconscious Bias	Foundational concepts on unconscious bias	Recognize ways your identity impacts how you see the world.
		Assess the relationship between identity and unconscious bias.
		Identify ways unconscious bias may be at work in everyday life, relationships, and in the healthcare setting.
		Locate and use tools to help you recognize your own unconscious bias.
		Reflect on ways you can mitigate your own unconscious bias.
Module 2: Microaggressions	Information on the types and effects of microaggressions	Recognize micro-messages.
		Define and identify micro-inequities and micro-affirmations.
		Identify ways micro-inequities may be at work in everyday life, relationships, and in the healthcare setting.
		Reflect on ways you can mitigate your use of micro-inequities and expand your use of micro-affirmations.
Module 3: Solutions	Tangible tips for how to mitigate personal biases	Recognize issues relating to bias in the environment around you.
		Define equality and equity.
		Define advocacy.
		Explain the concept of being an ally
		Summarize the ideas of privilege and power.
		List methods for Speaking Up against bias.
Module 4: Self-Awareness	Consider where unconscious biases originate and how they impact relationships	Define self-awareness.
		Outline the importance of self-awareness.
		List methods for becoming more self-aware.
		Test their own self-awareness.
		Write about bias in their own lives.
Module 5: Bias in Health	Bias and disparities in healthcare, emphasizing the “why” – “Why does it matter that we all have unconscious biases?”	Explore historical health disparities among marginalized groups
		Read date related to disparities in health care and other elements of daily life
		Summarize issues uniquely experienced by women in medical treatment
		List reasons to be aware of bias in medical education and treatment

### Measures

To access the course, each participant used or created a user profile for MyNRMN. Data include profile data (race, gender, ethnicity, degrees, career level, education level, degrees, having a parent/guardian attend college), course engagement data (start date, completion date, # of modules), and NRMN network size. After each module, participants responded to a reflection prompt(s). For this paper, we focused on the reflection prompts for four of the modules as these items are applicable to all course participants. These reflection prompt questions include: “What do ‘unconscious or implicit biases’ mean to you in your lived experiences?” (Module 1); “What are microaggressions and how are they related to unconscious bias?” (Module 2); “What are the different ways you can be an ally or advocate in your professional circles?” (Module 3); and “Describe two techniques that you believe will help self-awareness.” (Module 4). We did not include the Module 5 reflection for this analysis since it was focused on healthcare, which may not resonate with all course users.

### Data analysis

The study had a waiver of informed consent documentation. We provided descriptive information on demographic characteristics of participants who started and completed the course. We downloaded reflection responses for Modules 1–4. Through an iterative process, each question had a codebook generated from the data. Open and axial coding was conducted for the qualitative responses. Matrices were created for each response and the linkage to each axial code. Summaries were created and exemplary quotes were selected for each axial code. These were reviewed by the research team. We also conducted Chi-square and Fisher Exact tests, where appropriate, and unadjusted logistic regression models to compare the course completion rate by demographic characteristic. Analysis of the course data was approved by the North Texas Regional Institutional Review Board.

## Results

The sample was restricted to users between July 17, 2020 and May 26, 2021 (*n* = 977). Among these users, 518 people responded to at least one reflection question from each of the five modules. Not all data elements for registration were required entry points; therefore, a majority of registrants did not complete gender, ethnicity, education, parent with a college degree, degree, or career level. Most participants identified as White and were in a mentee role. Approximately one out of three participants had at least one connection in the NRMN network (Table 2). Less than half (*n* = 406, 42%) of users completed all five modules and 43% (*n* = 422) of users did not complete any module (Table 3). Completion proportions were higher for males compared to females, Asians compared to other racial groups, Non-Hispanics compared to Hispanics, post-bacs compared to other educational degree programs, staff compared to other career groups, and persons with a network size of 1–3. Statistically significant differences were observed for completion rate within each demographic category. Logistic regression models revealed that people with missing demographic data were at higher odds to complete the training compared to males (OR = 4.0, 95% CI 2.6, 6.1), Non-Hispanics (OR = 4.1, 95% CI 3.0, 5.5), people from “other” education/career levels (OR = 5.1, 95% CI 3.7, 7.0), people with parents without a college degree (OR = 5.4, 95% CI 3.4, 8.5), people with a bachelor’s degree (OR = 4.1, 95% CI 2.3, 7.5), and faculty (OR = 4.1, 95% CI 2.9, 6.0). Additionally, people who identified as Asian were at higher odds to complete the training compared to people who identified as White (OR = 1.8, 95% CI 1.2, 2.6). Mentees and members were at higher odds of completing the training compared to mentors (OR = 2.4, 95%CI 1.8, 3.2; OR = 4.1, 95% CI 2.7, 6.4; respectively). People with zero people in their network (OR = 2.4, 95% CI 1.4, 4.0) and 1–3 people in their network (OR = 4.8, 95% CI 2.7, 8.5) were at higher odds of completing the training compared to people with a network greater than 3.

**Table 2:** Participants in unconscious bias training, United States 2020–2021 (*n* = 977)

	Initiated Registration N (%)	Completed All Trainings N (%)	Proportion Completed	*P*-value^**a**^
**Gender**				< 0.0001
Male	133 (13.6)	36 (8.9)	27%	
Female	358 (36.6)	83 (20.4)	23%	
Other	1 (0.1)	1 (0.3)	100%	
Prefer Not to Report	10 (1.0)	3 (0.7)	30%	
Missing	475 (48.6)	283 (69.7)	60%	
**Race**				0.0032
White	557 (57.0)	238 (58.6)	43%	
Black/AA	118 (12.1)	39 (9.6)	33%	
Asian	121 (12.4)	69 (17.0)	57%	
AI/AN	14 (1.4)	6 (1.5)	43%	
NH/PI	3 (0.3)	1 (0.3)	33%	
Two or More	28 (2.9)	8 (2.0)	29%	
Other	43 (4.4)	16 (3.9)	37%	
Prefer Not to Report	71 (7.3)	24 (5.9)	34%	
Missing	22 (2.3)	5 (1.2)	23%	
**Ethnicity**				< 0.0001
Non-Hispanic	352 (36.0)	87 (21.4)	25%	
Hispanic	62 (6.4)	6 (1.5)	10%	
Prefer Not to Report	27 (2.8)	7 (1.7)	26%	
Missing	536 (54.9)	306 (75.4)	57%	
**Role**				< 0.0001
Mentee	447 (45.8)	218 (53.7)	49%	
Mentor	400 (40.9)	113 (27.8)	28%	
Member	113 (11.6)	70 (17.2)	62%	
Missing	17 (1.7)	5 (1.2)	29%	
**Education/Career Level**				< 0.0001
Undergraduate	36 (3.7)	13 (3.2)	36%	
Postbac	9 (0.9)	7 (1.7)	78%	
Graduate	80 (8.2)	13 (3.2)	16%	
Postdoc	47 (4.8)	8 (2.0)	17%	
Other (currently working/Faculty/Not in school or formal program)	311 (31.8)	70 (17.2)	23%	
Missing	494 (50.6)	295 (72.7)	60%	
**Parent with College Degree**				< 0.0001
No	127 (13.0)	27 (6.7)	21%	
Yes	324 (33.2)	67 (16.5)	21%	
Missing	526 (53.8)	312 (76.9)	59%	
**Degree**				< 0.0001
Bachelors or Less	65 (6.6)	16 (3.9)	25%	
Masters	75 (7.7)	11 (2.7)	15%	
Doctoral	282 (28.9)	60 (14.8)	21%	
Missing or Not Applicable	555 (56.8)	319 (78.6)	57%	
**Career**				< 0.0001
Faculty	223 (22.8)	49 (12.1)	22%	
Staff	15 (1.5)	5 (1.2)	33%	
Postdoc	27 (2.8)	5 (1.2)	19%	
Administrator	26 (2.7)	4 (1.0)	15%	
Other	99 (10.1)	23 (5.7)	23%	
Student	3 (0.3)	0	0%	
Scientist	16 (1.6)	3 (0.7)	19%	
Missing or Not Applicable	568 (58.1)	317 (78.1)	56%	
**Network Size**				< 0.0001
0	658 (67.4)	257 (63.3)	39%	
1–3	230 (23.5)	130 (32.0)	57%	
More than 3	89 (9.1)	19 (4.7)	21%	

^a^Fisher Exact or Chi-Square test

**Table 3:** Module Completion, United States 2020–2021 (*n* = 977)

Module	% Completed
**0**	422 (43%)
**1**	61 (6.2%)
**2**	47 (4.8%)
**3**	17 (1.7%)
**4**	24 (2.5%)
**5**	406 (42%)

### Unconscious bias in your lived experience

Following completion of Module 1, participants (*n* = 518) were asked about their view on unconscious and implicit biases relative to their lived experience. The five most prominent themes were action, judgment, external factors, stereotypes, and unintentional.

While unconscious biases are difficult to detect, participants reflected that they want to be more aware of unconscious and implicit bias concepts generally. To that end, this theme was largely described as taking on some sort of ***action*** within their lives. This point was described by 25% (*n* = 127) of the participants. Participants described needing to “*check*” themselves and realize they contribute to or have unconscious biases. Actions included reflection, awareness, and continual work in this area. As one participant stated, unconscious bias is a.

“*lens of self-awareness to evaluate my interactions and decisions*.”

Another participant wrote,

“*It [unconscious bias] means that I need to check myself to make sure that, as much as possible, I am evaluating actual information, not perceptions*.”

The next theme, ***judgment****,* described unconscious bias as a judgment or assumption toward others (*n* = 94,18%). These judgments may be due to personal characteristics such as race/ethnicity, nationality, religion, gender, or other visual features. Essentially, these judgments are made from preconceived ideas. As one participant stated,

*“It means that even if you don’t think you are being biased, you are still judging people based on how they related to your experiences, and you are not seeing them for who they are.”*

An additional theme was ***external factors***, meaning unconscious bias can be influenced by external forces (*n* = 71, 14%; i.e., social norms, media, or environment). Participants described unconscious bias as being attributed to their upbringing, socialization, or even being “*hardwired*.” As some participants stated,

*“They [unconscious biases] are part of the way we were raised, who we socialized with, what we watched in the media all influence this type of bias.”*

*“I believe unconscious bias can be the behaviors that I unintentionally learned from people I viewed as role models (parents, teachers, friends, etc.) and how they behaved, what they believed, and especially how they treated others.”*

Similar to the concept of judgments, ***stereotypes*** were discussed as a manifestation of unconscious bias (*n* = 63, 12%). Among some respondents, they equated unconscious bias as having stereotypes or assumptions about other people or groups of people. For example,

*“Unconscious bias is an automatic tendency for people to perceive others, situations, and events in stereotypical ways which affect our understandings, actions and decisions unconsciously.”*

Other respondents described that because of their personal identity, people stereotyped them. As one participant described,

*“Growing up as the daughter of first-generation [B] lack college graduates raised in the south, my parents continually taught me that bias and stereotypes exist and to excel beyond the expectations of the stereotype and to have sympathy for those who thought less of me because of my skin color.”*

Finally, one of the key themes present was that unconscious bias was ***unintentional*** (*n* = 62, 12%). Respondents described these biases as unintentional, not realizing they had them, or it being in “*auto-mode*.” As one participant stated,

*“It means that there is the potential that I unintentionally make choices that negatively impact people around me.”*

### Microaggressions

Following completion of Module 2, participants reflected on what microaggressions are and how it is related to unconscious bias. Responses (*n* = 445) had sub-categories for what are microaggressions, how do microaggressions present, what are the effects of microaggressions, and the relationship between microaggressions and unconscious bias.

The most prominent description of microaggressions (*n* = 420, 94%) was that they are harmful communications, actions, or behaviors (*n* = 314). A smaller proportion of respondents linked microaggressions to stereotypes based on social constructs (*n* = 13) or messages of bias (*n* = 36).

*“Microaggressions are the small actions and statements directed at people for their physical and cultural differences. These are the physical actions and verbal remarks that personify unconscious bias.”*

At times, these harmful actions could be packaged in a compliment – a hidden insult (*n* = 34).

*“Microaggressions are subtle comments made to a marginalized group of people. These compliments [microaggressions] are often stated as if they are compliments.”*

Participants also described microaggressions in relation to consciousness (*n* = 254; 57%). Most stated that microaggressions manifest unconsciously (*n* = 180), meaning the action was not done intentionally to cause harm. As one respondent stated,

*“Microaggressions are subtle. Often the offender is not aware of the action and consequence.”*

Fewer people described microaggressions as both intentional and unintentional (*n* = 74).

Participants shared the potential effects (*n* = 182, 41%), primarily that microaggressions create negative interactions or disruptive environments (*n* = 110). Participants noted that microaggressions may also invalidate or devalue people (*n* = 72).

*“Microaggressions and unconscious bias are subtleties that can devalue the identity and experiences of a person.”*

Finally, participants reflected on the connection between microaggressions and unconscious bias (*n* = 285, 64%). Most often, participants stated that microaggressions were rooted in unconscious bias, meaning they were a product of bias or rooted in stereotypes (*n* = 200). One participant provided an example of this relationship,

*“Microaggressions can be rooted in our unconscious biases; for instance, if we unconsciously think that a woman’s place is in the home, we might assume that a mother would care for her child more than a father would for his.”*

Other participants stated microaggressions and unconscious bias were related closely to one another due to the unintentional nature (*n* = 64).

*“Microaggressions are typically unconscious remarks that offend the receiver in some way. It is related to unconscious bias because most are unaware that they are doing it.”*

### Advocacy and allyship

After Module 3, participants (*n* = 388) reflected on ways they could be an ally or advocate Responses centered around four key themes: immediately acting (*n* = 321, 83%), reflection (*n* = 180, 46%), improving the organizational culture (*n* = 116, 30%), and individual-level allyship (*n* = 171, 44%).

The most commonly reported ***immediate action*** to be an ally or advocate involved responding to a problem when witnessing discrimination or bias from others (*n* = 294). As one participant reflected,

*“I think I struggle with speaking up when I hear racist and derogatory comments. I usually can identify when something like this has been said, and I usually just freeze up. This module has helped me to recognize that I can be an ally to standing up to racism and discrimination when I witness it. I cannot continue to be passive, but must take a stand. I really appreciated all of the practical examples this module gave of how to respond in these situations. If I am an ally, it will help to open the door for others to be allies as well!”*

An additional action participants stated they could take was educating themselves and others around issues of bias when engaging as an ally (*n* = 75) and prompting others to think about their biases by holding them accountable (*n* = 22). Some participants stated they may report occurrences of discrimination or bias to higher authorities (*n* = 12). One participant shared the following steps for action:

*“The first step would be to notice your actions and of those around you. If you feel someone is making derogatory remarks or can potentially construe hurtful feelings, address it politely by speaking to the person responsible and making them aware of the impact or effect of their words/actions. If the person disagrees, you may not interact with them, and become an ally to the targeted person as he or she probably feels betrayed. In workplace and other institutions, you may even report the person of derogatory behavior and some official action may be taken to address the issue. One must also appreciate the courage of an ally to support in difficult times as it helps in making better relationships and restores trust in the society/community.”*

Participants also shared that ***reflection*** was a key component of being an ally. Reflection involves awareness of injustices (*n* = 109), self-awareness of one’s own biases (*n* = 54), listening to understand others’ experiences (*n* = 33), and reflecting on how they can take on the social responsibility as an advocate or ally (*n* = 18). As one participant shared,

*“... I can be vigilant to notice these things. I can practice what I will say when these situations arise. I can reflect on my own unconscious biases to ensure that I’m not the one committing microaggressions, and I can be honest with myself and work to improve when I fail in this goal.”*

At a broader level, participants stated they could be an ally or advocate by ***improving the organizational culture*** (*n* = 116), which included promoting equity (*n* = 35; e.g., justice and fairness), inclusivity (*n* = 31; e.g., shared power and decision-making), and diversity (*n* = 9; e.g., diverse voices). A participant described improving organizational culture in academia,

*“Personally, I feel as though gaining confidence in speaking up will be critical, although this oftentimes feels difficult given the hierarchical structure of academia. Instead, supporting inclusive opportunities where all individuals’ identities and voices are recognized and respected (and where there is power in numbers) might be a first logical step.”*

Changes in organizational culture also involved creating safe spaces (*n* = 23) and setting expectations for what is and is not acceptable (*n* = 42).

*“One of the biggest ways I can help is by using my privilege to help others. This can include calling people out, questioning the basis of their statements, or making clear what behaviors are unacceptable in my presence.”*

Finally, participants shared they could be an ***ally or advocate at the individual level*** (*n* = 171), which involved supporting initiatives and people (*n* = 75), using their privilege to support others (*n* = 61), questioning others when bias occurs (*n* = 28), establishing new relationships (*n* = 26), and modeling these ally behaviors for others (*n* = 16). One participant reflected on using privilege and supporting others to overcome injustice,

*“If you have privilege, especially white privilege or male privilege, speak up and use your voice and resources to advocate for those who can’t themselves. Or not necessarily who can’t, but who aren’t being listened to due to the system.”*

### Actions for self-awareness

Following completion of Module 4, participants described two techniques they believed would help with self-awareness (*n* = 376). For these responses, three themes were the most prominent.

A majority of participants listed the need for ***reflection*** on their biases that may contribute to their actions (*n* = 202, 54%). In other words, it was a way to identify their blind spots or “*self check*.” As one participant stated,

*“Taking the time to recognize why I have my judgments or why they are happening and identifying my blind spots.”*

Others stated an action for self-awareness of ***pausing and breathing*** so that they could slow down and choose their response in a situation (*n* = 125, 33%). An example was stated by one participant as,

“*taking the time to self-assess before I respond to a stimulus*.”

The third most common action was ***observing themselves***, which included observing how they react in their environments and noting their reactions (*n* = 107, 28%). An example of self-observation is,

“*being more aware of the behaviors and words I use in certain situations.”*

### Key themes

Key themes were observed across the module reflections. Throughout these module reflections, participants were able to first understand the concept of unconscious bias and connect this *unintentionality and implicit nature* of bias to microaggressions. Moreover, in the allyship and action for self-awareness, participants shared *reflection* as a key strategy as well as other individual action steps, such as pausing and observation. Finally, throughout the modules, participants reflected on how unconscious bias is rooted in socialization, manifests in negative environments, and requires organizational change. These concepts reflect the *systemic nature of bias* in our society.

## Discussion

This study described the implementation of an asynchronous course on unconscious bias delivered on the NRMN platform to reach mentors and mentees in STEMM. The assessment of the NRMN Unconscious Bias Course revealed the course reached a wide cross-section of people in STEMM and that a subset completed the entire course offering, thus demonstrating the feasibility of this approach. Moreover, course participants were able to reflect on the underpinnings of the course related to unconscious bias overviews, microaggressions, solutions, and allyship.

The NRMN Unconscious Bias Course reached a nationwide audience of mentees, mentors, and other persons interested in increasing diversity in healthcare, healthcare education, and biomedical research. A diverse sample participated in the course, and almost half of the registrants completed the five-part series. We found that people most likely to complete the training had incomplete profile data on the MyNRMN platform, likely reflecting new users who were registering specifically for this course. The social reckoning with racism in the Summer 2020 may have fueled the participation in this type of educational offering. Furthermore, a majority of the registrants were in a mentor or mentee role when participating in the course. Pairing didactic training related to bias with mentoring can further support the translation of concepts into action [29]. Moreover, as the public debate regarding the inclusion of critical race theory ensues in the K-12 school system, integrating courses on unconscious bias, diversity, and inclusion may be necessary for higher education trainees.

Inclusion of a qualitative reflection with the course modules permitted an examination of participant perspectives on unconscious bias and description of potential application to their lives. Participants shared that unconscious bias is a concept that they need to be aware of and realize they may be contributing to bias through judgements, norms, or stereotypes. Participants also identified the potential harms associated with microaggressions, while recognizing microaggressions manifest unintentionally. Based on the reflections shared, learners were able to move through the initial step of *acceptance* or recognition of unconscious bias existing [30]. Most importantly, participants recognized specific ways they can be allies or advocates in their professional lives, not only at an individual level (e.g., reflection), but within an institution or organization. Systemic racism is embedded within institutions of higher education and STEMM, and thus requires intentional action to dismantle [9, 31, 32]. Overall, the reflections from the participants in the NRMN Unconscious Bias Course aligned with developed learning objectives to disseminate actionable information on addressing unconscious bias in STEMM.

The current literature has produced debate regarding the utility of unconscious bias and diversity training for behavior change [22–24]. Recently, an interactive workshop on implicit biases was evaluated for medical students, residents, and faculty. The study found significant improvements in knowledge and perceptions on bias [33]. A workshop on implicit biases regarding Hispanic patients was tested among first year medical students, and found improvement in implicit association tests among some of the students [34]. As is needed for this study, future research should examine long-term behavioral impacts of the unconscious bias training to determine if this dosage of exposure translates into behavior change. Unfortunately, the ambiguity in the literature regarding the ability for unconscious bias training to produce meaningful behavior change may be attributed to the endpoints that are measured. For example, the Implicit Association Test is a common surrogate endpoint that has predictive validity for behavioral, judgment, and physiological measures, albeit small effect sizes [35]. In contrast, self-reported behavior or behavioral intentions measures used as endpoints are limited by social desirability bias. Cumulatively, these endpoints for bias training result in difficulty measuring the efficacy of training; however, we should not discount the need for these approaches. Moreover, evaluating the impact of training with other strategies for dismantling systemic bias is needed. Providing a comprehensive approach that goes beyond only bias training, such as mentoring and professional development, ensures we are mitigating bias more effectively [29]. The NRMN platform is uniquely situated to explore these potential synergistic effects of diversity, equity, and inclusion courses and access to culturally-responsive mentoring.

## Limitations

The examination of the reflection data from the NRMN Unconscious Bias Course shows that participants who completed the course gleaned the underpinnings of the course; however, pre- and post-test data were not collected to assess change in knowledge, attitudes, and beliefs due to the course. Moreover, it is of utmost importance to understand participants’ application of course lessons and if, and how these have resulted in meaningful changes in behavior, devoid of bias, in the long term. Thus, we will implement pre-post data collection for evaluation, and continue to collect longitudinal data to understand the long-term effect of the participants who have completed the course. Further, we did not analyze Module 5, Bias in Health Care, which focused on patient health and healthcare, which may not resonate with all course users. Nonetheless, we recognize this module would provide insights on participants’ perspectives regarding health care and disparities and plan to analyze this module in the future. An additional limitation of this analysis is that we cannot discern whether someone participated due to an organizational requirement vs. voluntary nature. The motivation influencing participation may introduce sampling bias to this study. Additionally, attrition bias occurred in the course with less than half of the original users completing all five modules. This may be due to time constraints to complete all five modules, or lack of interest to complete the course. As a result, persons who were most interested or familiar with unconscious bias may have been more likely to complete the course and respond to the reflective prompts. Finally, given the nature of the course materials, reflexivity was used in the qualitative coding of the reflection responses to attempt to remove researchers’ influence on the theme generation.

## Conclusion

The NRMN Unconscious Bias Course is accessible to a nationwide audience of not only individuals who can take the course but also organizations and institutions who can leverage the course and offer it to its members. Additionally, the uniqueness of this course is that it is within a virtual environment in which participants are able to engage in a suite of offerings such as mentoring, networking, and professional development. This study can guide future groups who develop courses related to unconscious bias or diversity to show potential avenues for disseminating such a course (i.e., in a virtual platform), considerations for assessment and reflection with course participants, and potential content that resonated with participants in the course.

## Data Availability

The datasets generated and/or analyzed during the current study are not publicly available because they are programmatic data from NRMN but are available from the corresponding author on reasonable request.
